# Submillimeter-Resolution PET for High-Sensitivity Mouse Brain Imaging

**DOI:** 10.2967/jnumed.122.264433

**Published:** 2023-06

**Authors:** Han Gyu Kang, Hideaki Tashima, Hidekatsu Wakizaka, Fumihiko Nishikido, Makoto Higuchi, Miwako Takahashi, Taiga Yamaya

**Affiliations:** 1 Department of Advanced Nuclear Science, National Institutes for Quantum Science and Technology, Chiba, Japan; and; 2 Department of Functional Brain Imaging, National Institutes for Quantum Science and Technology, Chiba, Japan

**Keywords:** submillimeter resolution, preclinical PET, depth of interaction, in vivo mouse brain imaging

## Abstract

PET is a powerful molecular imaging technique that can provide functional information on living objects. However, the spatial resolution of PET imaging has been limited to around 1 mm, which makes it difficult to visualize mouse brain function in detail. Here, we report an ultrahigh-resolution small-animal PET scanner we developed that can provide a resolution approaching 0.6 mm to visualize mouse brain function with unprecedented detail. **Methods:** The ultrahigh-resolution small-animal PET scanner has an inner diameter of 52.5 mm and axial coverage of 51.5 mm. The scanner consists of 4 rings, each of which has 16 depth-of-interaction detectors. Each depth-of-interaction detector consists of a 3-layer staggered lutetium yttrium orthosilicate crystal array with a pitch of 1 mm and a 4 × 4 silicon photomultiplier array. The physical performance was evaluated in accordance with the National Electrical Manufacturers Association NU4 protocol. Spatial resolution was evaluated with phantoms of various resolutions. In vivo glucose metabolism imaging of the mouse brain was performed. **Results:** Peak absolute sensitivity was 2.84% with an energy window of 400–600 keV. The 0.55-mm rod structure of a resolution phantom was resolved using an iterative algorithm. In vivo mouse brain imaging with ^18^F-FDG clearly identified the cortex, thalamus, and hypothalamus, which were barely distinguishable in a commercial preclinical PET scanner that we used for comparison. **Conclusion:** The ultrahigh-resolution small-animal PET scanner is a promising molecular imaging tool for neuroscience research using rodent models.

## Visual Abstract

**Figure:**
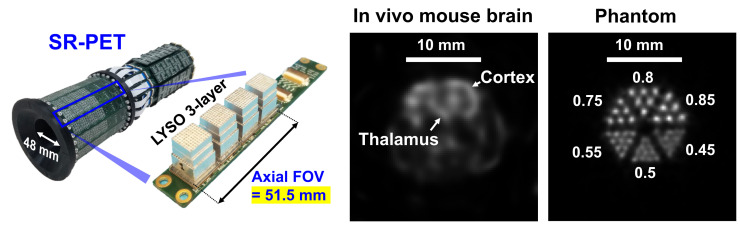

In vivo imaging of rodent models is crucial to understand the underlying mechanisms of various human diseases such as cancer (1*,*^2^) and neurodegenerative diseases (3*,*^4^); in turn, this understanding can lead to the discovery of new drugs for human diseases. PET has been playing a distinctive role in preclinical research as a molecular imaging tool that provides spatiotemporal information on biochemical processes in living animals (5). Small-animal PET imaging is particularly useful to discover and assess specific biomarkers of cancer and neurodegenerative diseases at a measurable picomolar level.

However, the spatial resolution of commercial PET scanners (6–9) has been limited to around 1.0–1.3 mm, which cannot distinguish the components of small objects such as the mouse brain, whose substructural organs (e.g., cortex and thalamus) are located near one another on the order of a few hundred micrometers. Even for the state-of-the art small-animal PET scanners developed since late 2020 by laboratories (10–14) and companies (15–18), the spatial resolutions are still around 0.8–1.2 mm and not good enough to identify small mouse-brain structures in detail, making it difficult to assess subtle alterations of mouse brain activity in neurodegenerative disease models.

One major factor limiting the spatial resolution of small-animal PET scanners is the crystal pitch, which typically ranges from 1.2 to 1.6 mm (19). A second factor is depth-of-interaction (DOI) information, which can preserve the spatial resolution in a small-ring-diameter geometry in which resolution blurring by the photon noncollinearity effect is relatively small (20). Intercrystal scattering (ICS) is a third factor that degrades spatial resolution by assigning the line of response to the wrong crystal positions, especially in finely pixelated crystal arrays (21).

In this study, we developed an ultrahigh-resolution small-animal PET scanner that addresses these technical issues to provide submillimeter resolution in a 51.5-mm-long axial coverage. A silicon photomultiplier (SiPM)–based staggered 3-layer DOI detector (22) was used to build a PET scanner for in vivo submillimeter imaging of the rodent brain.

## MATERIALS AND METHODS

### Submillimeter-Resolution Small-Animal PET Scanner

**FIGURE 1.**
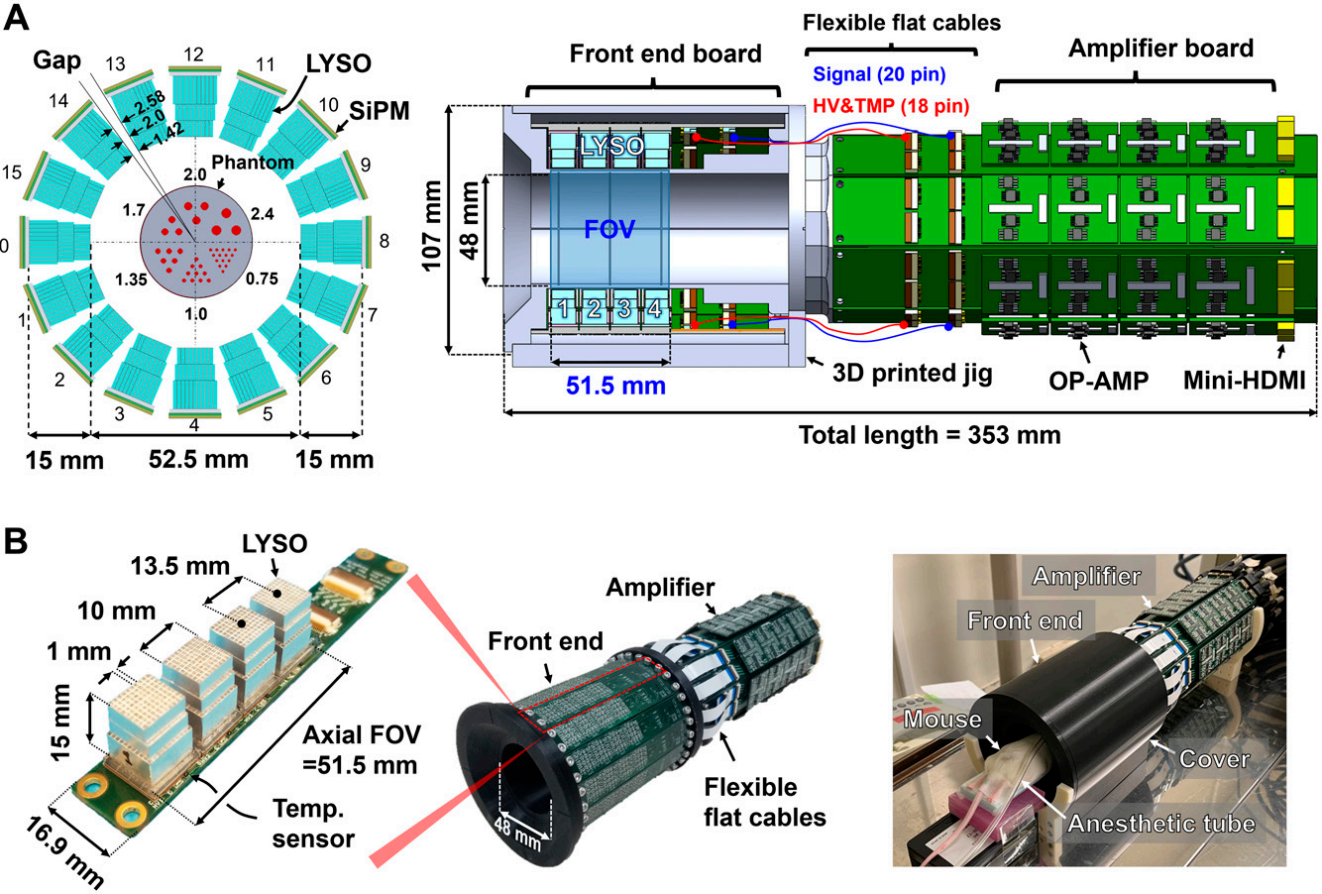
(A) Schematic drawings of SR-PET scanner in front and side views. (B) Photographs of SR-PET scanner with one front-end board, and in vivo mouse imaging setup with PET scanner. HDMI = high-definition multimedia interface cables; HV = high-voltage; OP-AMP = operational amplifier; TMP = temperature.

The submillimeter-resolution small-animal PET scanner (referred to here as the SR-PET) has 4 rings of 16 DOI detectors each, resulting in a 52.5-mm inner diameter and a 51.5-mm axial field of view (FOV) (Fig. 1A). Each DOI detector (22) consists of a 3-layer lutetium yttrium orthosilicate (LYSO) crystal array (EPIC Crystal), a 1-mm-thick acrylic light guide, and an SiPM 4 × 4 array (S14161-3050HS-04; Hamamatsu Photonics K.K.) (Fig. 1B; Supplemental Video 1; supplemental materials are available at http://jnm.snmjournals.org). The 3-layer LYSO crystal array consists of a first (10 × 9), second (10 × 10), and third (11 × 11) layer stacked in a staggered configuration with an offset of crystal half pitch in the radial and axial directions to encode the DOI information in the crystal map (Supplemental Fig. 1). The crystal thicknesses of the first, second, and third layers are 4, 4, and 7 mm, respectively. The LYSO crystals (0.9 × 0.9 mm cross section) are optically isolated using a 0.1-mm-thick BaSO_4_ powder layer, resulting in a 1-mm crystal pitch. Each crystal layer is optically coupled using epoxy glue (refractive index, 1.52; EPO-TEK 301-1 [Epoxy Technology]). The 3-layer LYSO crystal array, light guide, and SiPM are optically coupled using room temperature vulcanizing silicon rubber (refractive index, 1.45; KE420 [Shin-Etsu Chemical Co., Ltd.]). The top surface of the first crystal layer is covered by 2 layers of Teflon (Nichias) tape with a total thickness of 0.2 mm. The radial gap distances between the detector blocks are 1.42, 2.0, and 2.58 mm, for the first, second, and third layers, respectively (Fig. 1A). Four LYSO crystal arrays are mounted on the SiPM with a spacing of 13.5 mm in the axial direction (Fig. 1B). A cylindric lighttight cover is used to block external light.

For the SiPM signal readout and amplifications, custom-made front-end and amplifier boards are used (Supplemental Fig. 2A). A front-end board has 4 SiPMs, which are mounted with a central pitch of 13.5 mm in the axial direction (Supplemental Fig. 2B). An SiPM bias voltage of +41.0 V (overvoltage, 3.2 V) is applied to the common cathode (Supplemental Fig. 2C). Sixteen anode signals of each SiPM are reduced into 4 positional signals using a resistive network (Supplemental Fig. 2D). The positional signals from the front-end board are transferred to the amplifier boards through 10-cm-long flat, flexible cables. A timing signal that also carries the energy information is generated by summing the 4 positional signals. Each amplifier board consists of 4 add-on amplifier boards, and each add-on board can process 10 analog signals from 2 DOI detectors (Supplemental Fig. 2E).

The positional signals are amplified using a low-power quad-channel amplifier (OPA4684IPWT; Analog Device). The timing signal is fed to a fast amplifier (AD8000; Analog Device), and then a pole-zero-cancellation circuit is used to obtain fast pulse rise (26 ns) and decay (144 ns) times (Supplemental Fig. 2F). The temperature of each SiPM is monitored by a sensor (LM94023; Texas Instruments) attached near the SiPM. The SiPM ambient temperature is maintained at 26°C ± 0.4°C by an air conditioner in the experimental room to minimize the SiPM gain drift because of the temperature change. No temperature compensation technique was used for the SiPM since the maximum variation of the ambient temperature was within only ±0.4°C. The amplified SiPM analog signals from each amplifier board are transferred to a custom-made interface board via four 3-m-long high-definition multimedia interface cables (Supplemental Fig. 3). Subsequently, the SiPM signals are sent to a custom-made data acquisition system (23) via four 1.8-m-long radiofrequency shielded cables (Hewtech). The 320-channel 8-bit data acquisition system is used to digitize the SiPM analog signals with a sampling rate of 50 MSPS and an integration time of 250 ns.

The list-mode PET data are acquired in the singles mode and then stored on the hard disk of a desktop personal computer. Subsequently, the prompt coincidence events are identified using coincidence processing software with a coincidence window of 10 ns. The random coincidence events are recorded by a delayed coincidence window with a time offset of 260 ns.

The normalization data are obtained for 72 h by rotating a 0.16-MBq ^68^Ge–^68^Ga line source (diameter, 2 mm; length, 260 mm) using a motor stage (SGSP-80YAW; Sigmakoki) with a rotating radius of 22.5 mm.

### Image Reconstruction

For image reconstruction, analytic and iterative algorithms are used—namely the 2-dimensional (2D) filtered-backprojection (FBP) and list-mode 3-dimensional (3D) ordered-subset expectation-maximization (OSEM) algorithms. A voxel size of 0.25 × 0.25 × 0.25 mm^3^ and a matrix size of 200 × 200 × 206 are used for image reconstruction. For the 2D FBP algorithm, oblique sinograms are rebinned into direct sinograms using a single-slice rebinning algorithm (24) and then are reconstructed with a gap-filling method (22) that does not degrade the spatial resolution (Supplemental Fig. 4).

For the list-mode 3D OSEM algorithm, the detector response function modeling and normalization factors are incorporated into the system matrix. The system matrix is computed using the Siddon ray tracing algorithm with 5 subdivided crystal positions for each crystal layer (22). Eight subsets and 10 iterations are used unless otherwise specified. 3D gaussian image domain blurring (IDB) is incorporated during the image reconstruction to smooth the reconstructed images. The iteration number and IDB kernel size (i.e., full width at half maximum) are determined by a visual check, depending on the imaging object (Supplemental Table. 1). For PET data correction, normalization and random correction are performed. Scatter and attenuation corrections are not used. For all reconstructed PET images, contrast is adjusted only for the maximum level whereas the minimum level is set to zero, without any adjustment.

### Physical Performance Evaluation

Evaluation of the physical performance of the SR-PET scanner was based on the National Electrical Manufacturers Association (NEMA) protocol. To evaluate the spatial resolution and sensitivity, a NEMA ^22^Na point source (Eckert and Ziegler Isotope Products) with a diameter of 0.25 mm and an activity of 0.26 MBq was used.

The energy resolution and coincidence timing resolution were evaluated with the ^68^Ge line source positioned at the center of the FOV. A Voronoi diagram was applied to a crystal map to extract the energy information on individual crystals (22*,*^25^). Subsequently, a global energy spectrum was generated by summing all energy spectra of 64 DOI detectors after photopeak alignment for individual crystals. Then, system energy resolution was calculated by the ratio of full width at half maximum to the photopeak position without applying SiPM saturation correction (22). A global timing spectrum was obtained from the time stamp information of the 64 DOI detectors with an energy window of 400–600 keV after time skew correction (23).

The axial sensitivity profile was obtained by translating the ^22^Na point source with a step distance of 0.5 mm (crystal half pitch) from −26.5 to 26.5 mm. PET data were taken for 1 min for each axial position. ^176^Lu intrinsic radioactivity and the positron branching ratio of the ^22^Na source (i.e., 0.91) were considered for the sensitivity calculation.

Spatial resolution was measured using the ^22^Na point source from the center to the 15-mm radial offset position with a step distance of 2.5 mm (Supplemental Fig. 5A). In addition, spatial resolution was measured at different axial offset positions of 6.25, 13.5, and 19.75 mm, with an interval of 6.25 mm corresponding to the half pitch of the ring (Supplemental Fig. 5B). List-mode PET data were reconstructed using a 2D FBP algorithm without any gap-filling method. Iterative algorithms can artificially enhance spatial resolution for a point source in air, especially with extremely high iterations (26). Therefore, we chose 10 iterations for OSEM, where the radial full-width-at-tenth-maximum improvement plateaued and the radial full-width-at-half-maximum improvement had not yet plateaued (Supplemental Fig. 6). A line profile was extracted from the reconstructed PET image, and the full widths at half maximum and tenth maximum were then evaluated. An energy window of 440–560 keV was used, and the ^22^Na point source diameter, 0.25 mm, was not subtracted from the spatial resolution.

Count rate performance was evaluated with a 70-mm-long cylindric NEMA mouselike phantom (diameter, 25 mm). A 60-mm-long tubing source containing ^18^F solution (initial activity, 18.2 MBq) was inserted into the 3.2-mm-diameter hole of the phantom. PET data were acquired for 1 min every 30 min, until the activity decreased to 0.02 MBq. The true, scatter, random, and noise-equivalent count rates were calculated with energy windows of 250–750, 350–650, 400–600, and 440–560 keV, respectively (22).

To evaluate the recovery coefficient, spillover ratios, and uniformity, a NEMA NU4 image-quality phantom was filled with ^18^F-FDG of 1.7 MBq and then scanned for 180 min. An energy window of 400–600 keV was used. For the OSEM algorithm, an IDB kernel size of 1.25 mm was used without applying any postprocessing filter. For the FBP algorithm, the gap-filling method was applied, followed by 3D gaussian postprocessing filtering with a kernel size of 1 mm. The recovery coefficient, spillover ratios, and uniformity were calculated from the axially summed images with a 10-mm slice thickness.

### Resolution Phantom Imaging

The spatial resolution of the PET scanner was evaluated with 3 resolution phantoms in which the center-to-center distance of each rod was twice the rod diameter (Supplemental Fig. 7). First, a modified Ultra-Micro Hot Spot Phantom (Japan Radioisotope Association) (22) containing ^22^Na gel (0.77 MBq) was scanned for 60 min at the center of the PET FOV. The modified Ultra-Micro Hot Spot Phantom had 6 rod sectors (rod diameters of 0.75, 1.0, 1.35, 1.7, 2.0, and 2.4 mm) and an axial length of 8 mm (Supplemental Fig. 7A). The number of coincidence events was about 22 million. Image reconstruction used 10 iterations, a 0.5-mm IDB kernel size, and a 440- to 560-keV energy window. The reconstructed transverse PET images were projected in the axial direction with an 8-mm thickness, thereby producing an axially summed image. In addition, the effect of the energy window on spatial resolution was also investigated with various energy windows (250–750, 350–650, 400–600, and 440–560 keV), as ICS events can be discriminated on the basis of pulse height information with the 3-layer DOI detector (22*,*^25^).

The same-resolution phantom was also scanned using a commercial preclinical PET scanner (Inveon D-PET; Siemens) (8) for 10 min with an energy window of 350–650 keV to obtain 24 million true coincidence events. PET images were reconstructed using 2 different algorithms: 2D FBP and 2D OSEM. For 2D OSEM, 16 subsets and 4 iterations were used. A voxel size of 0.194 × 0.194 × 0.796 mm and a matrix size of 256 × 256 × 159 were used for Inveon PET image reconstruction.

Second, a SPECT rat phantom (22) having 6 different rod sectors (rod diameters of 0.7, 0.8, 0.9, 1.0, 1.2, and 1.5 mm) and a 12-mm axial length was filled with ^18^F of 2.1 MBq (Supplemental Fig. 7B). The SPECT rat phantom was placed at the center of the PET FOV and scanned for 60 min. PET images were reconstructed using OSEM with 10 iterations, a 0.5-mm IDB kernel size, and a 440- to 560-keV energy window. The transverse PET images were projected in the axial direction with a 12-mm thickness to generate a summed image.

Lastly, a PET mouse phantom having 6 rod sections (rod diameters of 0.45, 0.5, 0.55, 0.75, 0.8, and 0.85 mm) and a 10-mm axial length was filled with ^18^F of 1.1 MBq (Supplemental Fig. 7C). The PET mouse phantom was placed at the center of the PET FOV and then scanned for 60 min. For OSEM image reconstruction, 50 iterations and a 440- to 560-keV energy window were used. The reconstructed transverse PET images were axially projected with a thickness of 10 mm (i.e., 40 slices) to generate a summed image.

For quantitative evaluation of the spatial resolution, the valley-to-peak ratio (VPR) was calculated from the line profiles of each rod sector. Subsequently, resolvability (22*,*^27^) was calculated for each rod sector as follows:

$$\text{Resolvability}=\frac{N_{\text{Rayleigh}}}{N_{\text{Total}}}\times 100\%,$$

where $N_{\text{Rayleigh}}$ is the number of line profiles with VPR below the Rayleigh criterion (i.e., a VPR of 0.735) (22*,*^27^) and $N_{\text{Total}}$ is the total number of line profiles for each rod sector. The effects of IDB kernel sizes on SPECT rat and PET mouse phantom images were also investigated.

### In Vivo Animal Imaging

For metabotropic glutamate receptor imaging of a mouse brain, a 7-MBq dose of ^18^F-FITM (28)—a radioligand for metabotropic glutamate receptor 1—was administered to a conscious 9-wk-old, 20-g male nude BALB/cSlc-nu-mouse via the tail vein. Forty minutes after the injection, the mouse was anesthetized with 1.5%–2.0% isoflurane and underwent PET for 30 min. The 3D OSEM image reconstruction used 20 iterations, a 1.25-mm IDB kernel, and a 440- to 560-keV energy window.

For glucose metabolism imaging of a mouse brain, ^18^F-FDG with a radioactivity dose of 7 MBq was administered to a conscious 7-wk-old, 30.5-g male Slc:ddY mouse via the tail vein. The mouse was then allowed to move freely inside a cage for 30 min without any anesthesia so as to induce metabolic trapping of the radiotracer, reflecting cerebral glucose metabolism in an awake condition (29). Next, the mouse was anesthetized using isoflurane to minimize motion artifacts during the PET scan, and a 30-min PET data acquisition was done 34 min after injection. After this imaging session, an additional scan of the same mouse using the Inveon PET scanner was initiated 70 min after radiotracer injection and lasted 30 min. The Inveon PET data were reconstructed using 3D OSEM followed by maximum a posteriori estimation with 16 subsets, 2 iterations, a 350- to 650-keV energy window, and a matrix size of 256 × 256 × 159. The numbers of prompt coincidence events were 20 million for the Inveon PET scanner and 24 million for the SR-PET scanner. After the series of 2 PET scans, the mouse was imaged in a preclinical CT scanner (CosmoScan GX; Rigaku) using a 70-kV tube voltage and an 80-μA tube current to obtain anatomic information. The CT images had a voxel size of 0.24 × 0.24 × 0.24 mm^3^ and a matrix size of 256 × 256 × 512. To register the PET and CT images, PMOD software (version 3.4) was used. For all mouse brain PET images, the central 25 × 25 mm^2^ square was cropped and displayed.

For glucose metabolism imaging of a rat brain, ^18^F-FDG with a radioactivity dose of 12.3 MBq was administered to a conscious 8-wk-old, 283-g male Sprague–Dawley rat via the tail vein. The rat was anesthetized with isoflurane, and a 50-min brain scan with the SR-PET was done 140 min after ^18^F-FDG injection. Ten iterations, a 1.25-mm IDB kernel size, and an energy window of 440–560 keV were used for OSEM image reconstruction.

All animal experiments were conducted in accordance with the animal experiment guidelines of the National Institutes for Quantum Science and Technology after being approved by the local ethical committee of the institute.

## RESULTS

### Physical Performance

**FIGURE 2.**
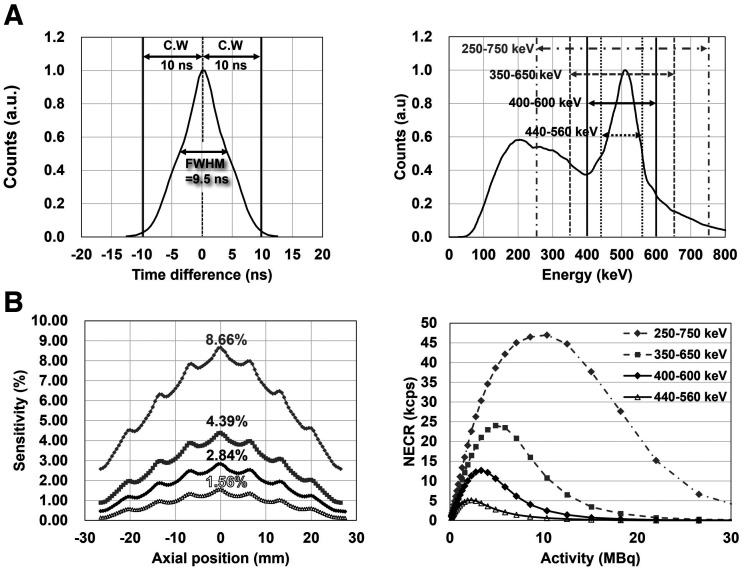
(A) Global timing and energy spectra. (B) Axial sensitivity profiles, and noise-equivalent count rate curves with energy windows of 250–750, 350–650, 400–600, and 440–560 keV. a.u. = arbitrary units; C.W = coincidence window; NECR = noise-equivalent count rate; ns = not statistically significant.

The system coincidence timing resolution was 9.5 ns, and the system energy resolution was 24.3% (Fig. 2A). The axial sensitivity profiles had symmetric distributions, with peak axial sensitivities of 8.66%, 4.39%, 2.84%, and 1.56% for energy windows of 250–750, 350–650, 400–600, and 440–560 keV, respectively (Fig. 2B). The peak noise-equivalent count rate decreased from 46.9 to 5.14 kcps as the energy window was narrowed from 250–750 to 440–560 keV (Supplemental Fig. 8; Supplemental Table 2).

The average radial resolutions from center to the 10-mm radial offset position for the axial offset positions of center and 6.25, 13.5, and 19.75 mm were 1.00 ± 0.16, 0.91 ± 0.05, 0.98 ± 0.12, and 0.91 ± 0.04 mm with FBP (Supplemental Table 3) and 0.58 ± 0.12, 0.61 ± 0.19, 0.58 ± 0.19, and 0.56 ± 0.19 mm with OSEM (Supplemental Table 4).

**FIGURE 3.**
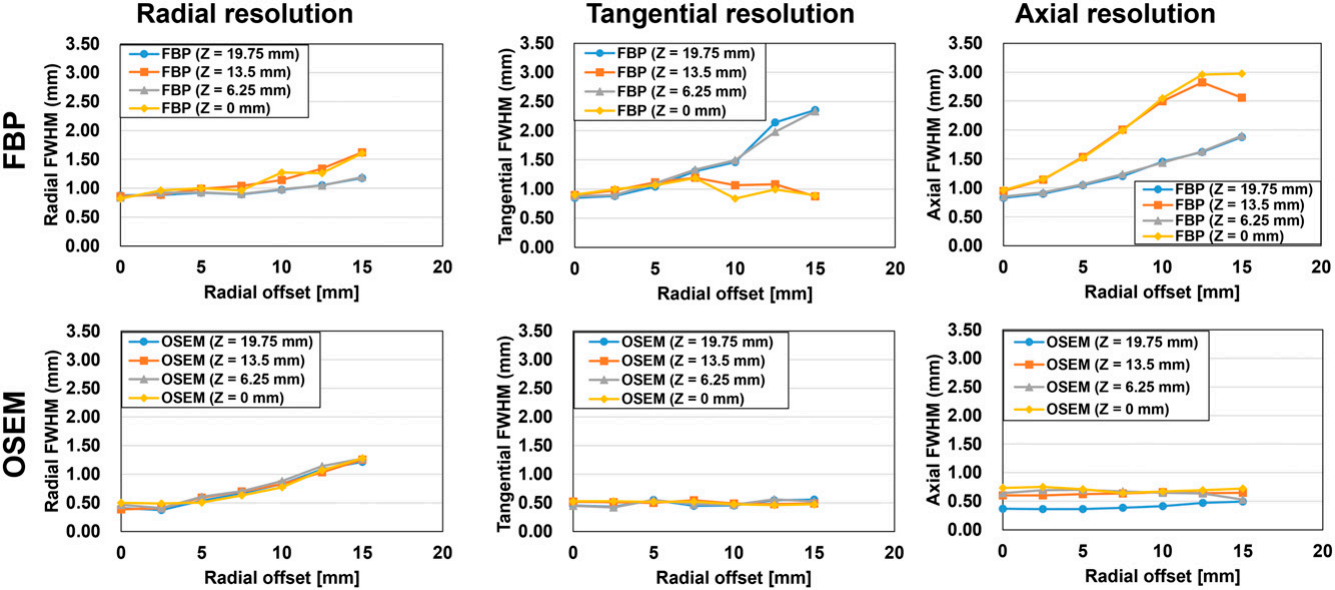
Spatial resolution measurements with ^22^Na point source at different radial and axial offset positions using energy window of 440–560 keV. Radial, tangential, and axial resolutions are shown with FBP (top) and OSEM using 10 iterations (bottom).

Spatial resolution was dependent not only on radial position but also axial position (Fig. 3). The axial resolution with FBP was degraded at an axial offset of 0 and 13.5 mm, where there were no direct lines of response. Resolution degradations in the axial and tangential directions were effectively reduced using the OSEM algorithm, which accounted for the geometric factors. However, axial resolution was degraded for positions near the axial center because of parallax error in the axial direction. Even with high iterations of over 10 (Supplemental Fig. 9), the radial resolution improvement for the radial offset of 10 and 15 mm was less dramatic than the radial offsets of within 5 mm because of parallax error.

The NEMA NU4 phantom images (Supplemental Fig. 10) and analysis results (Supplemental Table 5) indicate good image quality in terms of recovery coefficient, uniformity, and spillover ratios.

### Resolution Phantom Images

**FIGURE 4.**
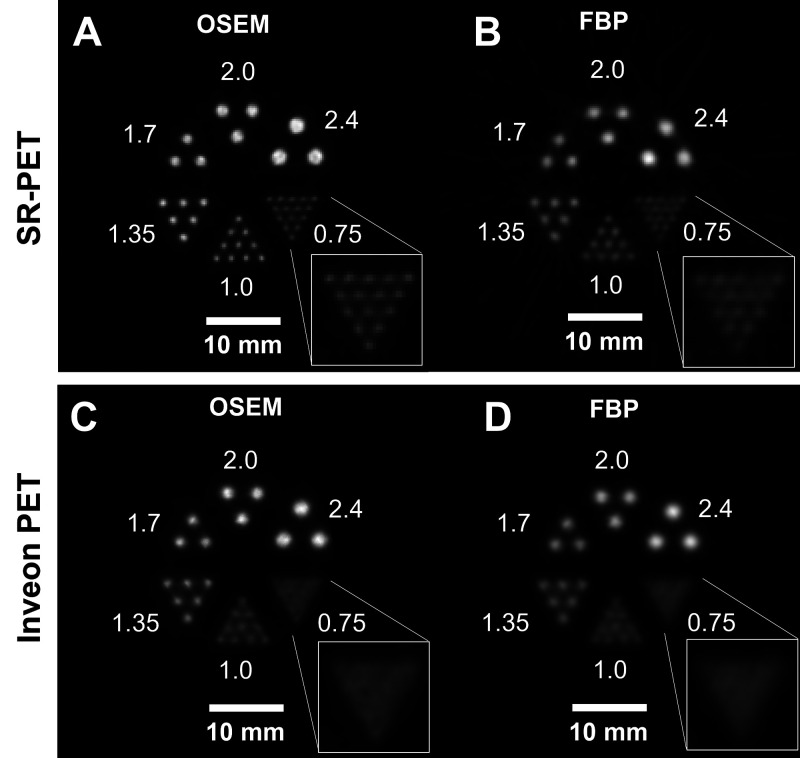
(A and B) Reconstructed PET images of modified Ultra-Micro Hot Spot Phantom with SR-PET using OSEM (A) and FBP (B). (C and D) Reconstructed PET images of same phantom with Inveon PET using OSEM (C) and FBP (D). Rod diameters are 0.75, 1.0, 1.35, 1.7, 2.0, and 2.0 mm. Inset represents 0.75-mm rod sector.

The 0.75-mm rod structures of the modified Ultra-Micro Hot Spot Phantom were resolved with an average VPR of 0.543 ± 0.065 and 100% resolvability (Fig. 4A). The 0.75-mm rod structure could also be resolved using the 2D FBP algorithm with an average VPR of 0.775 ± 0.079 with a resolvability of 33.3% (Fig. 4B). In contrast, the Inveon PET could not resolve the 0.75-mm rod structure of the same phantom with FBP (Fig. 4C) and OSEM (Fig. 4D) because of the limited spatial resolution of 1.0–1.3 mm. For the SR-PET scanner, spatial resolution improved as the energy window was narrowed from 250–750 keV to 440–560 keV (Supplemental Figs. 11 and 12) because of decreased ICS events at the expense of sensitivity (Supplemental Table 6).

For the reconstructed OSEM image of the SPECT rat phantom, all rod patterns from 0.7 to 1.5 mm were resolved 100% (Supplemental Table 7).

**FIGURE 5.**
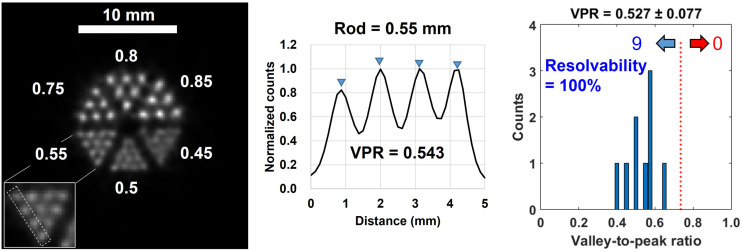
(Left) Reconstructed image of mouse phantom obtained with SR-PET for 60 min after cropping 25 × 25 mm central square. (Center) Line profiles of 0.55-mm rod sectors obtained as marked by white dotted boxes of insets. (Right) VPR histogram for diameter of 0.55 mm. Numbers of line profiles with VPRs under and over 0.735 are shown at left and right arrows, respectively.

To resolve structures smaller than 0.6 mm, 50 iterations were used for the PET mouse phantom (Supplemental Fig. 12), thereby resolving the 0.55-mm rod pattern with an average VPR of 0.527 (resolvability, 100%) (Fig. 5). However, the resolvability for the 0.5- and 0.45-mm rod patterns was only 55%, and 8%, respectively (Supplemental Table 7).

### In Vivo Rodent Brain Images

**FIGURE 6.**
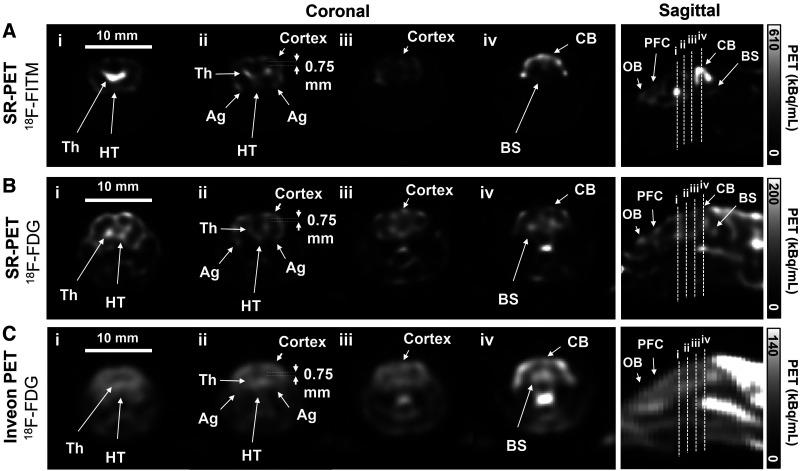
(A) Mouse brain images of ^18^F-FITM (7 MBq) obtained for 30 min with SR-PET 40 min after injection. (B) Mouse brain images of ^18^F-FDG (7 MBq) obtained for 30 min with SR-PET 34 min after injection. (C) Mouse brain images of ^18^F-FDG (7 MBq) obtained for 30 min with Inveon D-PET 70 min after injection. For all images, 25 × 25 mm central square was cropped and displayed. Coronal images were selected from 4 different slices as indicated in sagittal images by dotted lines (i–iv). Ag = amygdala; BS = brain stem; CB = cerebellum; HT = hypothalamus; OB = olfactory bulb; Th = thalamus; PFC = prefrontal cortex.

For rodent brain imaging, an IDB kernel size of 1.25 mm was used to smooth the images while maintaining submillimeter resolution (Supplemental Figs. 14 and 15). Representative coronal mouse brain PET images at 4 different planes 1 mm apart were selected for visual display (Fig. 6). High accumulations of ^18^F-FITM in the thalamus and cerebellum of the nude mouse could be observed in the PET images (Fig. 6A; Supplemental Video 2). The cortex, thalamus, hypothalamus, and amygdala of the nude mouse were also well delineated. In the sagittal image, the olfactory bulb and prefrontal cortex were well identified.

^18^F-FDG images of the mouse brain allowed clear identification of the cortex, thalamus, and hypothalamus, which were located close to one another, with only 0.5- to 0.75-mm separations (Fig. 6B). In addition, the amygdala, whose position was near the cortex, could be identified. In contrast to the SR-PET, the Inveon PET could barely resolve brain structures in the same mouse because of the low spatial resolution (Fig. 6C).

**FIGURE 7.**
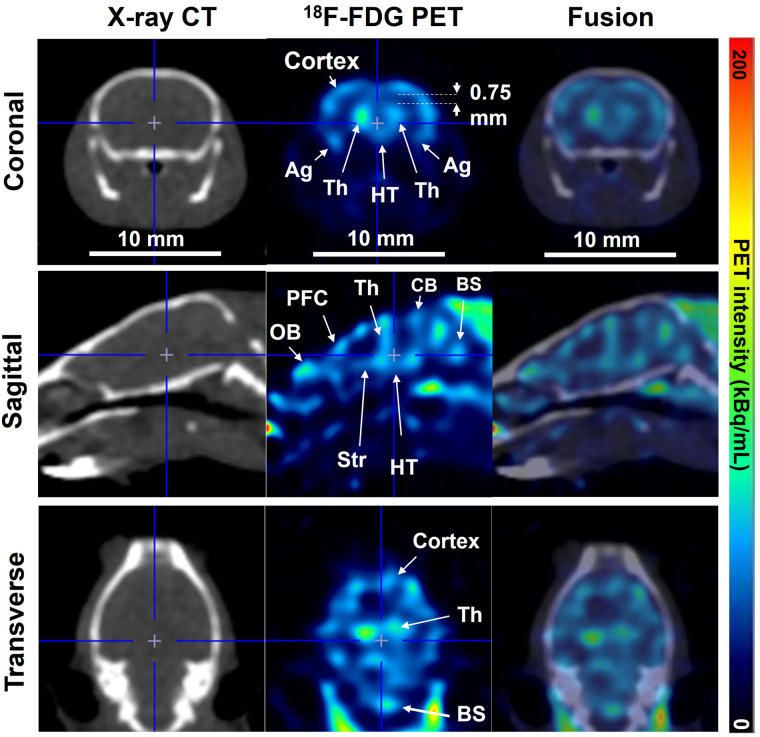
Mouse brain images obtained with ^18^F-FDG PET, CT, and their fusion in coronal (top), sagittal (middle), and transverse (bottom) planes. For all images, 17 × 17 mm central square was cropped for image display. Ag = amygdala; BS = brain stem; CB = cerebellum; HT = hypothalamus; OB; olfactory bulb; Str = striatum; Th = thalamus; PFC = prefrontal cortex.

^18^F-FDG SR-PET images of the mouse brain coregistered well with CT images in the coronal, sagittal, and transverse planes (Fig. 7). Details of brain structures within the cranial bone were delineated well. In addition, glucose metabolism in the rat brain was clearly visualized with SR-PET (Supplemental Fig. 16).

## DISCUSSION

We developed the SR-PET using 3-layer DOI detectors that can achieve submillimeter spatial resolution in a 51.5-mm-long axial coverage (Fig. 1). Several factors limit the spatial resolution of a small-animal PET scanner (20*,*^30^), including crystal pitch, crystal decoding error, sampling error, parallax error, photon noncollinearity, positron range (20), and ICS events (21). The fine crystal pitch (1 mm) combined with the staggered 3-layer DOI configuration can substantially minimize sampling error (31*,*^32^) and parallax error (30). The staggered 3-layer DOI detector allowed us to construct the PET scanner with small gaps between detector blocks, thereby minimizing loss of projection information because of detector gaps (32). The crystal decoding error (30) was substantially reduced using a diffusive reflector material (BaSO_4_ powder) (25). The small ring diameter (52.5 mm) of the PET scanner minimized spatial resolution degradation caused by photon noncollinearity (20). Furthermore, parallax error caused by the small ring diameter was effectively reduced by the 3-layer DOI information. Finally, ICS events could be rejected with the narrow energy window of 440–560 keV (Supplemental Figs. 11 and 12) since ICS events have relatively lower or higher pulse height than photoelectric events because of the light collection efficiency difference, which depends on the crystal layer (22*,*^25^*,*^33^). As a result, the SR-PET resolved the 0.55-mm rod structure with resolvability of 100% (Fig. 5). Previously, a spatial resolution of 0.55 mm was reported by a research group at UC Davis (34). However, the axial FOV (7 mm) was too short to cover the entire brain of a mouse, which is typically about 15 mm in length from the olfactory bulb to the cerebellum. A SPECT scanner using a clustered pinhole collimator dedicated to high-energy radiation (e.g., 511 keV) can resolve the 0.65-mm rod structure (35). However, the physical collimation technique demands an extremely high activity of over 20 MBq because of the poor sensitivity, 0.25%, whereas our small-animal PET scanner provides peak sensitivity of 1.56% even with a narrow energy window of 440–560 keV.

The closely adjacent cortex, thalamus, and hypothalamus were separately identified on in vivo mouse brain images with the SR-PET, whereas the Inveon PET scanner could not distinguish these structures, whose ^18^F-FDG distributions may show little change from those of the SR-PET scan because of washout (Fig. 6).

Although the SR-PET resolved the 0.55-mm rods in the resolution phantom, the resolution for in vivo mouse brain imaging was degraded to around 0.85 mm because of the reduced iteration number (from 50 to 20) (Supplemental Fig. 13) and increased IDB kernel size (from 0.5 to 1.25 mm) (Supplemental Fig. 14). Thus, we plan to optimize the in vivo imaging protocol (e.g., injection dose and scan time) to obtain more coincidence events so as to minimize resolution loss, especially by IDB kernel size. In addition, our next study will focus on integration of the PET scanner inside an ultrahigh-field MRI scanner to simultaneously obtain high-resolution morphologic information (10*,*^11^*,*^17^*,*^36^) while pushing the PET resolution limit using a high magnetic field (37*,*^38^). Finally, we plan to use the SR-PET scanner to detect subtle changes in cortical brain activity in mouse models of Alzheimer disease (39*,*^40^).

## CONCLUSION

We developed the SR-PET, a scanner that can provide a spatial resolution approaching 0.55 mm in a 51.5-mm-long axial coverage. The SR-PET can serve as a useful molecular imaging tool for translational neuroscience research using rodent models.

## DISCLOSURE

This work was supported by KAKENHI grants (20H05667 and 21K19936) and by grants from the Nakatani Foundation, the QST Future Laboratory Program, and the Directorate’s Fund Project given by Dr. Takashi Nakano. No other potential conflict of interest relevant to this article was reported.

## Notes

KEY POINTS **QUESTION:** Can we explore mouse brain function with submillimeter resolution using a highly sensitive small-animal PET scanner for neuroscience research? **PERTINENT FINDINGS:** The SR-PET provides a spatial resolution approaching 0.55 mm in a 51.5-mm-long axial coverage. Glucose metabolism in a mouse brain can be visualized in detail by resolving the cortex, thalamus, hypothalamus, and amygdala, which were barely distinguishable with a commercial preclinical PET scanner. **IMPLICATIONS FOR PATIENT CARE:** The SR-PET can serve as a useful molecular imaging tool for translational neuroscience research and discovery of new drugs for neurodegenerative diseases in rodent models.
